# Manipulated Spawning Along With an Extension of the Atlantic Salmon Broodfish Feeding Period Affect the Vitamin C, E, D, and K Status of Broodfish, Eggs, and First-Feeding Fry

**DOI:** 10.1155/anu/8874795

**Published:** 2025-11-11

**Authors:** Anne-Catrin Adam, Per Gunnar Fjelldal, Tom Hansen, Ernst Morten Hevrøy, Kristin Hamre

**Affiliations:** ^1^Feed and Nutrition, The Institute of Marine Research, Bergen, Norway; ^2^Reproduction and Developmental Biology, The Institute of Marine Research, Matredal, Norway; ^3^Mowi Feed, Mowi ASA, Bergen, Norway

## Abstract

The optimum period for feeding a broodfish diet combined with manipulated ovulation time, has recently been investigated for egg production capacity, as well as egg and juvenile quality in Atlantic salmon. Here, we report the status of vitamins C, E, D, and K in fish from the same experiment to ensure requirements were met. Two-sea-winter female broodfish were followed through a 17-month growth period, a starvation period on-land until ovulation, and offspring until first-feeding. Throughout all periods, the impact of 9 vs. 17 months of broodfish feed, and early (November), normal (December), and late (February) ovulation on vitamin status was monitored. Vitamin deposition increased with growth, with muscle depositing the highest amounts due to its size. Once the gonads matured, vitamins E, K, and D were similar to muscle, while C was higher. Livers had the highest C, E, and K concentrations, while D was comparable across tissues. During starvation, body stores of C, K, and D declined, while E remained high. All studied vitamins except for C followed the general nutrient deposition profile in unfertilized eggs, increasing from early to late ovulation. K1 was depleted as menaquinone-4 rose, suggesting possible conversion in broodfish and offspring. Ovulation time affected vitamin status more than feeding regime. Vitamin C, E, and most likely K and D levels in both feeding regimes covered the requirements for broodfish and first-feeding fry, regardless of ovulation treatment. However, both early and late eggs and juveniles were of inferior quality, suggesting factors beyond the vitamins examined influenced reproductive outcomes.

## 1. Introduction

Atlantic salmon (*Salmo salar*) spawns naturally in November/December, but in aquaculture, it is beneficial to have supply of high-quality eggs over longer periods of the year. Milt can be cryopreserved and is, therefore, easy to keep available all year round [1]. Ova, however, must be fresh and are made available through manipulation of ovulation time in female broodfish. Through the manipulation of day length, water temperature, and salinity, which regulate developmental speed of sexual maturation [2, 3], females commence to ovulate earlier or later than the natural ovulation period. Normal aquaculture practice is not to feed salmon broodstocks when transferred from sea cages to freshwater tanks in summer as salmon stop eating, analogous to the upward migration of wild salmon in the rivers. Therefore, the development of the ovary mainly depends on the reallocation of nutrients from somatic tissues of the female. Advanced ovulation may then give insufficient time to build up yolk stores with enough nutrients for sustaining the embryonic and larval developmental periods, while late ovulating females are deprived of food over a longer period, affecting fish welfare. Perhaps the gonads also continue to accumulate nutrients from body stores as indicated by egg dry matter in Fjelldal et al. [4]. Accordingly, manipulation of ovulation has been shown to affect the nutrient deposition in the eggs [5]. Skjærven et al. [6] reported that advanced ovulation impairs the optimal deposition of nutrients in the eggs, while delayed ovulation enhanced broodfish muscle catabolism, reflected in the nutrient composition of the eggs.

To produce eggs with higher quality and optimized nutrient composition, Atlantic salmon broodfish is fed specialized broodfish feeds for a limited period before transfer to freshwater. These feeds contain more fish oil and fish meal, and several micronutrients are supplemented above requirements, in contrast to the more cost-effective grow-out feeds [4, 7]. There is very little knowledge regarding the length of the feeding period with broodfish feed that will optimize egg production capacity, quality and nutrient composition. Traditionally, egg count or fecundity, along with egg size, are good parameters to assess egg producing capacity, while egg quality is commonly assessed by fertilization- and survival rates at eying, hatch and first-feeding [8, 9]. The present work is based on samples from the study Fjelldal et al. [4] that examined effects of different lengths of broodfish feeding, combined with manipulation of ovulation time, on egg production capacity and egg quality in Atlantic salmon. Reported feeding periods with broodfish feed were between 9 and 14 months [6, 10], between 6 and 15 months [11] and Fjelldal et al. [4] studied an extension of this period up to 17 months compared to 9 months. In the present study, the feed-dependent nutrient content was followed over time in female broodfish, eggs, and first-feeding fry, to investigate whether broodfish received optimal levels of the water-soluble vitamin C and the fat-soluble vitamins E, D, and K.

The antioxidant vitamins, C and E, are well known to protect against oxidation by free radicals that are generated through metabolism, tissue damage, infections, pollution, and seasonal changes in the environment in all organisms, including fish [12, 13]. The generic term vitamin E refers to different forms of tocopherols and tocotrienols, where α-tocopherol has the highest biological activity [14]. Vitamin E functions in redox balance and immunity [13, 15] as its primary function is to protect the organism against lipid oxidation through which vitamin E gets oxidized and then regenerated and/or spared by vitamin C [16, 17]. Vitamin C has shown to protect against vitamin E deficiency in a dose-dependent manner in first-feeding Atlantic salmon, particularly when a vitamin C deficiency induced a large drop in vitamin E in the liver [16]. For a wide range of fish species, both vitamin C and E have been shown to be necessary for fertilization and to play essential roles in reproduction [18, 19, 20, 21]. Increased levels of vitamin E upto 250 mg/kg in the feed improved both egg quality and fry survival in Atlantic salmon [22]. Other important functions of vitamin C for salmonids are attributed to detoxification, wound healing, and reproduction as reviewed by Dawood and Koshio [12] and Sandnes [23]. Vitamin D is best-known for its role in calcium (Ca) and phosphate (P) homeostasis in fish and other vertebrates [24, 25]. Vitamin D participates both in the absorption of Ca and P from the diet and water, and the deposition and resorption from bone through metabolization of the nonphysiologically active vitamin D3 into different active hydroxylated compounds, such as calcitriol. There is a well-known interaction between vitamin K and D, as both vitamins seem to act synergistically, while vitamin D increases urinary Ca excretion and vitamin K decreases it to reduce bone loss through Ca homeostasis [26]. The term vitamin K refers to a series of related compounds that function as cofactors in the synthesis of bone proteins like the Ca-binding osteocalcin, but also blood coagulation factors, and is, therefore, important for blood coagulation, bone metabolism, and growth control in fish [27, 28]. Vitamin K occurs naturally as phylloquinone (vitamin K1, mainly plant origin) and in different menaquinone forms (vitamin K2, mainly bacteria and algae origin). Menadione, which is the synthetic provitamin vitamin K3, is primarily used as an additional vitamin K source in animal feed. Vitamin K, and especially menadione is very unstable during the production and storage of feed [29], which makes requirement studies difficult to design.

This study reports tissue levels of the water-soluble vitamin C and the fat-soluble vitamins E, D, and K in Atlantic salmon broodfish, subjected to two feeding regimes with a specialized broodfish feed (17 months vs. 9 months) prior to ovulation. During a starvation period in autumn, fish were exposed to different environmental conditions to produce offspring at three ovulation times: early (November), normal (December), and late (February). It is part of a larger study where the aim was to investigate the feed-dependent status of many nutrients in female broodfish, eggs, and first-feeding fry to ensure both broodfish welfare and health of the next generation of farmed fish. Due to the huge amount of data, the study is reported in several articles: (1) biological performance [4]; (2) protein and amino acids; (2) lipids, fatty acids, cholesterol, and astaxanthin; (3) vitamin C and fat-soluble vitamins E, D, and K; (4) B-vitamins; (5) minerals (all except for [1] and [3] in preparation). The present work provides a unique data set on the vitamins C, E, D, and K during periods of different broodstock handling practices and the vitamin status of the offspring from female broodstock that ovulated early, normal, or late. Based on this framework, the following three hypotheses were tested: (1) a longer feeding period (17 months vs. 9 months) with broodstock feed enhances vitamin storage (C, E, D, and K) in broodfish tissues and subsequently in their eggs and offspring; (2) manipulated ovulation timing (early and late) compared to normal ovulation affects vitamin deposition in gonads and eggs due to differences in maturation and mobilization processes; (3) vitamin levels in both ovulating broodfish and first-feeding fry remain above deficiency thresholds (if known), supporting nutritional adequacy across treatments.

## 2. Materials and Methods

The vitamin data presented in this study originates from fish and feed samples from the broodfish experiment earlier described by Fjelldal et al. [4].

### 2.1. Experimental Set-up and Broodfish Body Indexes

**Figure 1:**
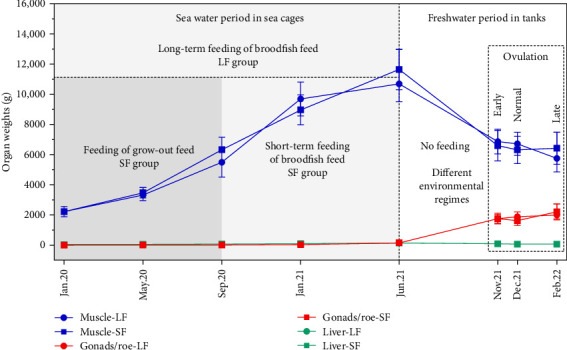
Experimental timeline and development of muscle, liver, and gonad weights in female Atlantic salmon broodfish during feeding, maturation, and ovulation. After a short or long-term feeding period with broodfish feed (Jan.20 until Jun.21), all groups were held in common garden in on-land tanks in three different temperature and photoperiod regimes to mature and ovulate either early (Nov.21), normal (Dec.21), or late (Feb.22). Organ weights are presented as mean ± SD for each feeding regime, but after freshwater transfer (Jun.21) not separated by different lines for each ovulation group for clarity purposes. After ovulation, fertilized eggs were incubated until first-feeding (not illustrated). Weight data reused from Fjelldal et al. [4].

In brief, female pit-tagged broodfish (Mowi Terningen, Mid-Norway) were initially stocked at The Institute of Marine Research (IMR), Matre (Western Norway) as yearling smolt (*n* = 2000) and randomly allocated to two 5 m × 5 m × 7 m sea cages under natural light. On January 15, 2020, these fish were randomly reallocated across four 5 m × 5 m × 7 m sea cages, with two cages per dietary treatment: either a commercial grow-out feed or a commercial broodfish feed (Mowi Feed, Valsneset, Mid-Norway), forming the short-term (SF; 9 months with broodfish feed) and long-term (LF; 17 months with broodfish feed) feeding groups, respectively, as shown in Figure 1 [4]. This provided two biological replicates per diet group during the seawater period. Body weight and health status were similar between groups throughout the trial [4], ensuring comparable initial conditions. From September 15, 2020, all four cages received the broodfish feed until transfer in June 2021. To minimize potential environmental differences between the original sea cages and ensure comparable conditions for all fish, nine maturing females from each of the four sea cages were randomly redistributed into a common garden setup in each of nine indoor tanks (three per ovulation group, in total 36 fish per tank; June 2021). The ovulation groups were subjected to three different environmental regimes combining photoperiod and temperature as earlier described [4] to produce three different ovulation times. Briefly, early ovulation was induced by a rapid drop in photoperiod (from continuous light to 8L:16D on July 13, 2021) combined with a cooling from 16 to 6°C by September 1, 2021. Normal ovulation followed simulated natural light and Matre River temperature profiles (max 16°C, min 4°C). Late ovulation was achieved by extended continuous light and elevated temperature (12–15°C) until January 3, 2022, before reducing both to stimulate final maturation. Salinity in all groups was gradually reduced from 25 ppt to freshwater (0.8 ppt) before stripping, and oxygen saturation was maintained above 80%. At time of ovulation in either November 2021 (early), December 2021 (normal), or February 2022 (late), eggs from all groups were fertilized with cryopreserved sperm from the same male and incubated at approximately 7°C until first-feeding at 828–865 degree-days (DD) post fertilization (Supporting Information 1: Table S1). Fjelldal et al. [4] reported that ovulation time but not feeding period (SF vs. LF group) had obvious effects on growth, egg production capacity, and egg quality. Body weight and, thus, muscle mass increased from the start of the experiment until June 2021 and decreased after transfer on-land (no feeding) and from early to late ovulation (Figure 1) [4]. At time of ovulation, hepatosomatic index was lower and gonadosomatic index higher in late ovulating females than in the other ovulation groups. All groups spawned a similar number of eggs per female, but the eggs and first-feeding fry of the late ovulation group were heavier than those from early and normal ovulating females.

### 2.2. Sampling Procedure for Vitamin Analysis

Tissue from female broodfish was sampled five times during the feeding period in sea cages and once at early, normal, or late ovulation (Figure 1). For broodstock analysis, six replicates were obtained per feeding regime, and 24 replicates were analyzed at each ovulation stage, comprising 12 from the LF group and 12 from the SF group (except for early ovulation, where only nine LF replicates were available), as detailed in Supporting Information 3. Prior to sampling, fish was uniformly fasted for 1 day to evacuate most feed from the intestine, stabilize blood, and tissue nutrient levels and minimize postprandial variation in metabolites and nutrient levels. Fish were then euthanized (0.5 g/L Finquel) followed by an accurate and forceful blow to the skull (percussive stunning) performed by trained personnel. Due to limited organ size during different periods of the experiment and other prioritized nutrient analyses, a few data points are missing from some of the vitamins. For sampling, homogenized liver and muscle (NQC) tissue, gonads/roe, unfertilized eggs, eggs at eyeing, (328–388 DD; Supporting Information 1: Table S1) and homogenized first-feeding fry (828–865 DD) were snap-frozen on dry ice and stored at −80°C to preserve vitamin stability. To ensure homogeneous nutrient distribution when analyzing eggs, excess liquid surrounding the eggs was drained away prior to freezing, and eggs were stored at −80°C before cryogenic grinding using the 6875D Freezer/Mill (SPEX SamplePrep, Metuchen, USA). This enabled thorough and uniform homogenization under cryogenic conditions using liquid nitrogen, as fresh, unfrozen eggs are difficult to process due to their structural integrity and surrounding membranes.

### 2.3. Feed Composition, Vitamin Analysis, and Data Treatment

The broodfish feed used in this experiment contains higher sums of eicosapentaenoic acid (EPA) and docosahexaenoic acid (DHA), and higher levels of some vitamins and minerals compared to the grow-out feed. Two batches of grow-out feed and three batches of broodfish feed were fed to salmon broodfish throughout the seawater period, following the feeding period design described in Fjelldal et al. [4] and shown in Figure 1. Vitamins, minerals, and amino acids were added to at least cover NRC requirements and levels are proprietary of Mowi Feed AS. Feed formulation and analyzed nutrient composition of grow-out and broodfish feed was first published in Fjelldal et al. [4] but is also provided in Supporting Information 1 (Tables S2 and S3). As the focus of this study was on the effect of feeding duration with two diets with large differences in nutrient levels, the smaller variation in vitamin content between batches of the same diet was not considered in this study.

Both feed (Table 1) and tissues were analyzed for α-, β-, γ-, δ-tocopherols and tocotrienols; vitamin K1 (phylloquinone), K2 (menaquinone derivates), and K3 (menadione; only analyzed in the feed); vitamin C (determined as ascorbic acid equivalents); vitamin D3 (cholecalciferol). Vitamin D3 was analyzed by high performance liquid chromatography (HPLC) [30, 31]. Levels of vitamin C were determined by measuring ascorbic acid using an HPLC method with electrochemical detection, as described in Mæland and Waagbø [32]. Prior to analysis, dehydroascorbic acid was reduced to ascorbic acid using dithiothreitol, which also stabilizes ascorbic acid, enabling quantification of total vitamin C that represents the biologically relevant pool of both reduced and oxidized forms. HPLC with fluorescence detection was used for phylloquinone (vitamin K1) and menaquinone (vitamin K2) analysis [33], and for α-, β-, γ-, δ-tocopherols and tocotrienols [28, 34]. To preserve vitamin stability at sampling, tissues were rapidly frozen on dry ice and stored at −80°C until analysis. All vitamin analysis methods are validated and performed at an ISO 17025:2025-accredited laboratory at IMR (Bergen, Norway). Analytical quality was ensured through regular participation in national and international proficiency testing schemes to assess accuracy, precision, and method uncertainty. Certified reference materials are analyzed at least annually to verify method performance, and in-house control materials are included in each analytical batch to monitor consistency and reliability.

Vitamin levels of all detected forms are reported for every tissue in Supporting Information 2 (Table S4). This data contains vitamin levels expressed as “<VALUE” that refers to concentrations below the limit of quantification. Vitamin levels in tissues are presented both as concentrations (mg/kg or µg/kg), and as total vitamin amounts (mg total or µg total) to provide a biologically relevant estimate of the absolute vitamin content per offspring stage or organ, beyond concentration alone. Total amounts were estimated by multiplying individual concentrations with individual weight data for liver, gonads, gutted weight, egg, or fry. After removing viscera and gonads from female broodfish, gutted weight was recorded and used as proxy for muscle weight. Total whole body vitamin amounts were estimated by summing the total amounts calculated for muscle, liver, and gonads.

### 2.4. Statistical Analysis

The vitamin levels in broodfish tissues were tested for interaction effects between sampling time (jan.20–feb.22) and feeding regimes (SF and LF) by factorial ANOVA, when assumptions of normality and homogeneity were met (Shapiro–Wilk and Levene's test). If not, the nonparametric Kruskar–Wallis ANOVA was used to test time effects. Differences between feeding regimes (SF vs. LF) at individual time points, tissues, and offspring stages were tested by Mann–Whitney *U* test. Interaction effects between ovulation time (early, normal, and late) and feeding regime history (SF and LF) were investigated in tissues and offspring stages using factorial ANOVA. When normality and homogeneity assumptions were not met, Kruskar–Wallis ANOVA was used to test ovulation time effects. For ANOVA tests, significant differences among group means were identified using Tukey's HSD or unequal N HSD post hoc tests, with adjustments for multiple comparisons as appropriate. Statistical significance was set at *p* <  0.05. All analyses were performed using TIBCO Statistica (version 13.4.0.14; TIBCO Software Inc, Palo Alto, CA, USA). Descriptive statistics for each vitamin are provided in Supporting Information 3 (Table S5).

## 3. Results

### 3.1. Vitamin Distributions Across Salmon Broodfish Tissues During Short- and Long-Term Feeding and Manipulated Ovulation Time

#### 3.1.1. Vitamin C

**Figure 2:**
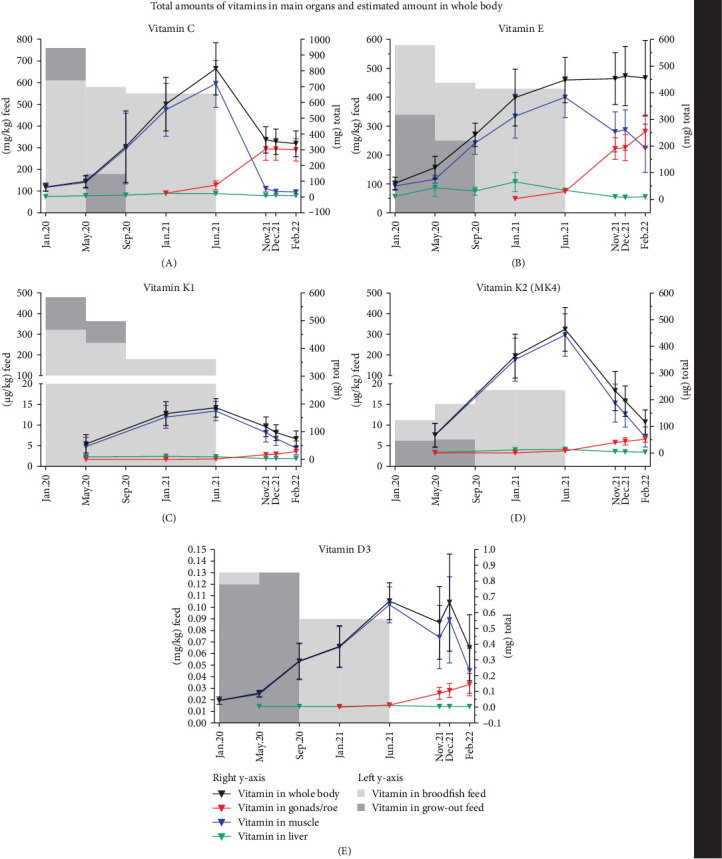
Total amounts of vitamin C, E, K1, K2, and D3 in broodfish tissues under different feeding regimes, during starvation and at early, normal, and late ovulation. (A–E) Dietary vitamin levels are illustrated in the background of each graph as gray shaded areas separating the levels for grow-out and broodfish feed (left y-axis: µg/kg feed or mg/kg feed) that were fed during the sea water period. The distribution of the total vitamin amounts in liver, gonads, and muscle is shown by colored lines (right y-axis: µg total or mg total). The sum of each vitamin in the three organs gives an estimate of the total vitamin in whole body. Absent gray shaded areas in the background mark the transfer from sea cages to on-land tanks and start of the starvation period (Jun.21) in three different groups that ovulate either early (Nov.21), normal (Dec.21), or late (Feb.22). Vitamin levels for each organ are presented as mean ± SD of all groups and not separated by either feeding regimes or ovulation groups for clarity purposes. Vitamin K is shown in µg/kg as per standard lab reporting. All other vitamins are shown in mg/kg.

**Figure 3:**
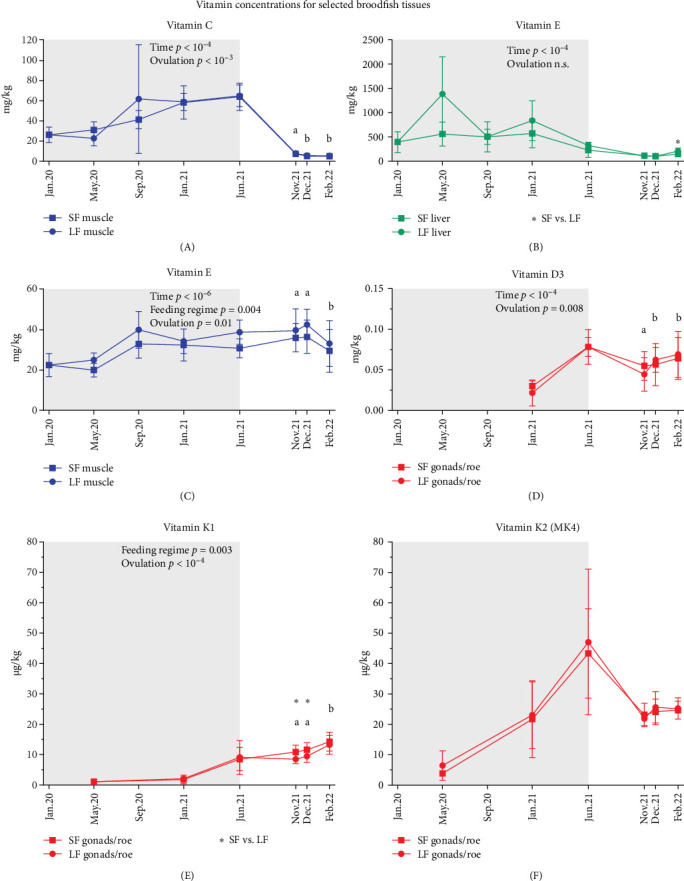
Development of vitamin C, E, D3, K1, and K2 concentrations for selected broodfish tissues before and around ovulation. (A–F) illustrate vitamin concentrations per tissue (mg/kg or µg/kg) for both the SF (short-term feeding) and LF (long-term feeding) groups following vitamin stores during the sea water period until Jun.21, and during starvation from Jun.21 until broodfish ovulated either early (Nov.21), normal (Dec.21), or late (Feb.22). Data is presented for selected tissues and vitamins to highlight the most relevant changes in vitamin concentrations. Vitamin levels for each tissue are presented as mean ± SD for each feeding regime but not separated by different curves after freshwater transfer (Jun.21) to separate groups by ovulation time. An asterisk marks a statistically significant difference between the feeding regimes at individual time points. Lowercase letters indicate statistically significant differences among ovulation groups. Group means and statistics are provided in Supporting Informaton 3: Table S5. Vitamin K is shown in µg/kg as per standard lab reporting. All other vitamins are shown in mg/kg.

The vitamin C content of the grow-out feed was 760 mg/kg at the beginning of the experiment. This content was greatly reduced to 180 mg/kg vitamin C when fed from May to September, while the broodfish feed levels fluctuated between 550 and 610 mg/kg vitamin C during the feeding period. Total vitamin C stores (mg total) in whole body increased from start of the experiment to freshwater transfer (jun.21) and then declined to nearly half by ovulation (Figure 2A). Muscle was the main storage for vitamin C at the end of the feeding period due to its mass (Figure 1), averaging 717 mg total vitamin C, but levels dropped sharply to 32 mg total as the gonads developed and became the main storage, reaching 298 mg total (Figure 2A and Supporting Information 3: Table S5). At time of freshwater transfer, gonads had also the highest vitamin C concentrations, averaging 463 mg/kg compared to 65 mg/kg in muscle and 153 mg/kg in liver (Supporting Information 1: Figure S1). Muscle concentrations were nearly depleted by the time of ovulation following starvation, averaging 5 mg/kg (Figure 3A and Supporting Information 3: Table S5).

Extended feeding with broodfish feed (LF vs. SF) had no significant effect on vitamin C levels in the liver, muscle, or gonads (Supporting Information 3: Table S5). At the time of ovulation, early ovulating females had lower vitamin C concentration in their liver compared to normal and late ovulating females (Supporting Information 1: Figure S1). In contrast, vitamin C concentrations in the muscles and gonads declined from early to late ovulation (Figure 3A and Supporting Information 1: Figure S1).

#### 3.1.2. Vitamin E

The reported results for vitamin E refer specifically to α-tocopherol, the form with the highest biological activity among all tocopherols and tocotrienols. The dietary levels of vitamin E were between 250 and 340 mg/kg in grow-out feed and dropped from 580 to 430 mg/kg in the broodfish feed during the feeding period (Table 1). Muscle was the main storage organ for vitamin E at the end of the feeding period, averaging 384 mg total (Figure 2B). During starvation, muscle levels dropped by about half, while estimated total body vitamin E remained relatively stable, accompanied by increasing amounts in the gonads with gonadal development from early to late ovulation (Figure 2B). Liver vitamin E concentrations were generally elevated during feeding compared to starvation. The highest mean concentration of 1385 mg/kg was observed in the LF group (may.20), whereas the SF group exhibited 560 mg/kg during the same period (Figure 3B). However, due to substantial inter-individual variability within the LF group, this difference did not reach statistical significance. During starvation, liver, and gonad vitamin E concentrations decreased (Figure 3B and Supporting Information 1: Figure S1), while muscle concentrations remained constant during periods of extensive muscle and gonad growth (Figure 3C).

Feeding regime had a significant effect on vitamin E concentrations in muscle tissue and estimated whole body stores during both feeding and starvation periods, with the LF group consistently exhibiting the highest levels (Figure 3C and Supporting Information 3: Table S5). Differences between feeding groups were also evident in late ovulating females, where both liver and gonad tissue in the LF group contained higher vitamin E levels compared to the SF group (Figure 3B and Supporting Information 1: Figure S1).

#### 3.1.3. Vitamin K

Vitamin K1 (phylloquinone) was the predominant form of vitamin K in all feeds, with lower levels in the broodfish feed compared to the grow-out feed throughout the feeding period (Table 1). Vitamin K2 forms (menaquinones MK4–MK10) were present at lower levels than K1 in all feeds. However, total K2 content was higher in the broodfish feed than in the grow-out feed (Jan.20–Sep.20). Supplemented synthetic menadione (vitamin K3) ranged from 0.8 to 6.4 mg/kg feed (800–6000 µg/kg), substantially higher than the combined total of vitamin K1 and K2, which ranged from 267 to 545 µg/kg (Table 1). Although MK7 and MK8 were relatively high in the feed, they were below the quantification limit or absent in the tissues (Supporting Information 3: Table S5).

Muscle contained the highest total amounts of both vitamin K1 and K2 (MK4) among the tissues, with MK4 being the predominant form despite its dietary level being substantially lower than K1 (Figure 2C,D and Table 1). Total whole body MK4 averaged 465 µg (Figure 2D), exceeding total K1, which averaged 186 µg at the end of the feeding period (Figure 2C and Supporting Information 3: Table S5). Total K1 and MK4 in muscle declined after the freshwater transfer, reflecting both a general reduction in muscle mass and decreased concentrations (Supporting Information 1: Figure S2). From early to late ovulation, total K1 and MK4 also decreased in both whole body and muscle (Figure 2C,D), consistent with a significant decline in muscle concentrations of vitamin K1 and MK4 (Supporting Information 1: Figure S2; *p* <  10^−4^). Gonadal MK4 concentrations were consistently higher on average than those of K1 at the end of the seawater period and throughout starvation until ovulation (Figure 3E,F and Supporting Information 3: Table S5). K1 remained stable during starvation, while MK4 decreased as the gonads matured (Figure 3E,F). From early to late ovulation, K1 increased significantly, whereas MK4 did not change.

Overall, the effect of ovulation time on the tissue K levels was stronger than the effect of feeding regime (Supporting Information 3: Table S5). Compared to early and normal ovulation, late ovulating females had significantly lower K1 and MK4 levels in their muscle (Supporting Information 1: Figure S2; *p* <  10^−4^), and significantly higher K1 levels in both liver (Supporting Information 1: Figure S2; *p* <  10^−4^) and gonads (Figure 3E). Before ovulation, few differences were observed between SF and LF groups. In late ovulating females, muscle K1 and MK4 concentrations were significantly higher in the SF group (Supporting Information 1: Figure S2; *p* <  0.05). Gonadal K1 concentrations were also higher in SF fed females that ovulated early or normal (Figure 3E; *p* <  0.05).

#### 3.1.4. Vitamin D3

Dietary levels of vitamin D were similar between grow-out and broodfish feed and ranged from 0.09 to 0.13 mg/kg (Table 1). Due to its large size, muscle was the primary storage tissue for vitamin D3, with total amounts averaging 0.65 mg at the end of the feeding period (Figure 2E). Unlike vitamins C, E, and K, tissue concentrations of D3 were relatively similar across liver, muscle, and gonads (Supporting Information 1: Figure S3 and Supporting Information 3: Table S5).

Feeding regimes had no significant effect on vitamin D3 status in broodfish. However, late ovulating females had significantly lower muscle D3 concentrations than early and normal groups (Supporting Information 1: Figure S3) after abstaining from food for the longest time. Gonadal D3 levels were highest in the normal and late ovulation groups, compared to the early group (Figure 3D).

### 3.2. Vitamins Stored in Eggs and First-Feeding Fry Dependent on Broodfish Feeding and Ovulation Time

#### 3.2.1. Vitamin C

**Figure 4:**
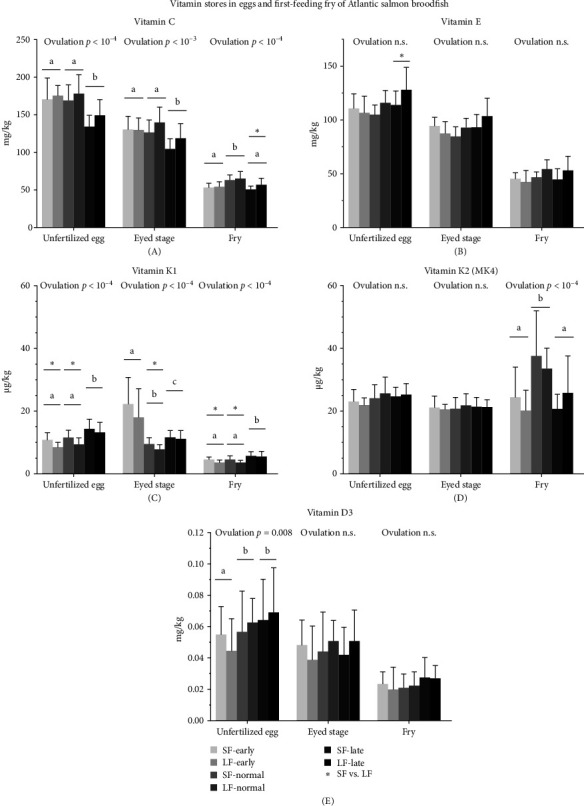
Vitamin C, E, K1, K2, and D3 levels in unfertilized eggs, eyed eggs, and first-feeding fry depending on the broodfish feeding regime and early, normal, or late ovulation. (A–E) illustrate vitamin concentrations (mg/kg or µg/kg) given as mean ± SD at each stage from early, normal, and late ovulating Atlantic salmon broodfish that have been fed a specialized broodfish feed for 17 (LF) or 9 months (SF) in sea water. Concentrations presented for unfertilized eggs correspond to the reported gonad concentrations at time of ovulation. Significant ovulation group differences are indicated by lowercase letters and an asterisk marks significant feeding regime differences at individual time points. Group means and statistics are provided in Supporting Informaton 3: Table S5. Vitamin K is shown in µg/kg as per standard lab reporting. All other vitamins are shown in mg/kg.

Vitamin C concentrations declined by more than 60%, from an average of 163 mg/kg in unfertilized eggs to 57 mg/kg in first-feeding fry across all groups (Figure 4A, Supporting Information 3: Table S5). For all offspring stages, vitamin C concentrations were significantly lowest at late ovulation compared to normal ovulation (Figure 4A).

In fry from the late ovulation group, the SF group had significantly lower vitamin C concentrations than the LF group (*p* <  0.05). Similarly, total vitamin C amounts (corrected for weight), were significantly higher in the LF group across all offspring stages from normal ovulation, compared to early and late ovulation groups (Supporting Information 1: Figure S4; *p* <  0.05).

#### 3.2.2. Vitamin E

Vitamin E stores, alike vitamin C, declined by more than 50% during the incubation period from an average of 113 mg/kg in unfertilized eggs to 48 mg/kg in first-feeding fry across all groups (Figure 4B). Total vitamin E amounts were higher in fertilized eggs and first-feeding fry from the late ovulation group compared to the normal ovulation group, although this difference was not significant in eyed stage eggs (Supporting Information 1: Figure S4).

Within the late ovulation group, unfertilized eggs from the LF group had significantly higher vitamin E concentrations than those from the SF group (Figure 4B; *p* <  0.05). Like for vitamin C, first-feeding fry with normal ovulation from the LF group contained significantly higher total vitamin E amounts than those from the SF group (Supporting Information 1: Figure S4; *p* <  0.05).

#### 3.2.3. Vitamin K

Solely vitamin K1 and the K2 vitamer MK4 were found in unfertilized eggs (Supporting Information 2: Table S4). At ovulation, egg vitamin K concentrations averaged higher for MK4 (21 µg/kg) than K1 (13 µg/kg) across all groups (Figure 4C,D). At first-feeding, vitamin K averaged 27 µg/kg for MK4 and 5 µg/kg for K1. The lowest K1 levels (3.6 µg/kg) were detected in first-feeding fry from the LF group with early and normal ovulation (Supporting Information 3: Table S5). Total vitamin K amounts (µg total) based on average egg and fry weights, also showed a decrease in K1 and an increase in MK4 from the egg to fry stage (Supporting Information 1: Figure S4), although this was not statistically evaluated. At first-feeding, MK4 averaged 0.004 µg total and K1 averaged 0.001 µg total per individual across all groups (Supporting Information 3: Table S5).

Feeding regime affected both concentration and total levels of vitamin K1 in unfertilized eggs, with significantly lower K1 concentrations in the LF group compared to SF in several comparisons (Figures 4C,D, and S4; *p* <  0.05). K1 concentrations were higher in late ovulated offspring compared to early and normal groups in unfertilized eggs and first-feeding fry, whereas the early group showed the highest levels in eyed eggs (Figure 4C). Feeding regime had no effect on MK4 concentrations or total amounts in the offspring. Similarly, ovulation time did not affect MK4 concentration in unfertilized and eyed eggs, but at first-feeding, fry from normal ovulated females contained significantly more MK4 than those from the early and late group (Figure 4D). Total MK4 levels increased from early to late ovulation in unfertilized and eyed eggs, with the highest amounts in first-feeding fry from the normal group (Supporting Information 1: Figure S4).

#### 3.2.4. Vitamin D3

Overall, vitamin D3 concentrations decreased from an average of 0.06 mg/kg in eggs to 0.03 mg/kg in fry, representing a reduction of nearly 60% from fertilization until first-feeding (Figure 4E).

The feeding regime had no effect on D3 concentrations or total amounts in the offspring. Deposition into gonads, indicated by significantly lower D3 concentrations in unfertilized eggs from the early ovulation group compared to normal and late groups, was no longer evident at the first-feeding stage (Figure 4E). When calculated based on average weights (mg total), total D3 amounts increased progressively from early to late ovulation across all stages (Supporting Information 1: Figure S4; *p* <  10^−4^).

## 4. Discussion

### 4.1. Muscle Was the Largest Vitamin Store During Broodfish Growth Until the Gonads Matured

When vitamin concentrations were multiplied by broodfish organ weights, muscle tissue represented the largest storage organ for vitamin C, D, E, and K during the sea water growth period. However, livers, compared to muscle and gonads, had much higher concentrations of the vitamins C, E, and K, but not D. Except for vitamin E, the estimated total vitamin stores in whole body decreased following the general decrease in fish weight during the starvation period. While muscle and liver mass decreased and gonads increased in size [4], the organ vitamin stores followed this pattern where the vitamins were transferred to the maturing gonads. Fjelldal et al. [4] reported a higher dry matter content of the roe (unfertilized eggs) from late ovulating females, which were deprived of feed for the longest period (approximately 9 months). That supports the assumption that the nutrient transfer from somatic tissues to the gonads continues independently of the hormone-driven maturing of the eggs for ovulation, as also indicated by the transfer of protein and lipid [46].

### 4.2. Vitamin Status in Broodfish

Both broodfish and grow-out feed contained very high vitamin C and E levels, which were reflected in the vitamin C and E levels in broodfish tissues and offspring. Liver vitamin E concentrations were highest in the LF group during the early growth period in spring (May 2020) when the dietary levels of vitamin E were also relatively high. Feeding the broodfish feed with 580 mg/kg vitamin E resulted in high average levels in the liver (LF group) with great variation between individuals that can be indicative for difficulties to cope such high dietary levels. It has been shown that salmon smolt in sea water oxidizes more and uses more of its stores of antioxidants, such as vitamin C and E during spring and summer time that increases the requirements up to 200 mg/kg vitamin E and 300 mg/kg vitamin C [35]. However, vitamin C and E at too high levels can turn pro-oxidative as shown for vitamin E by Hamre et al. [16]. At the highest concentration tested (300 mg/kg vitamin E) without any vitamin C supplementation, vitamin E was used (oxidized) but not regenerated, and the radical form accumulated [16]. In the present study, estimated whole body vitamin E stores and muscle tissue concentrations remained relatively stable during the starvation period, whereas total vitamin C stores and concentrations declined markedly across all tissue. This indicates an increased use of vitamin C to protect vitamin E stores that is in line with the results found earlier in Atlantic salmon [16]. Vitamin E, a lipid-soluble antioxidant, protects membranes from oxidative damage but needs vitamin C to regenerate once oxidized. The molecular basis of this antioxidant synergy involves the glutathione cycle, which helps recycle vitamin C and maintain vitamin E in its active form. In turn, oxidized glutathione can be reduced for example by NADPH. However, measurements of glutathione-dependent enzymes that sustain the reduced form of vitamin C, completing this protective cycle, were beyond the scope of this study. Overall, vitamin C and E concentrations in broodfish tissues remained relatively high throughout the whole experiment compared to the levels below 10 mg/kg (whole fish) that has been reported to cause a deficiency in first-feeding juveniles [36, 37].

All five batches of feed contained more vitamin K1 than K2 derivates, but menadione, the synthetic derivative K3 was present at 2–15-fold higher levels than the natural K vitamins. Vitamin K1 and the derivate MK4 were the main storage forms found in the tissues. Traces of the derivates MK7 and MK8 were found in broodfish tissue, but were not detected in the offspring, where only K1 and MK4 were detected. This shows a selectivity for storage and maternal transfer of these vitamin K forms.

Dietary vitamin D3 was nearly the same in every feed batch in the present study. Unlike the other vitamins, the deposition of D3, which is the main storage form for vitamin D, remained relatively stable and similar across tissues throughout the experiment, likely due to its lipid-soluble nature, which facilitates consistent storage and slower turnover. Dietary D3 was below the upper limit set by EU regulations [47] (1.5 mg/kg) and above the reported minimum requirements of 0.06 mg/kg [38] preventing deficiencies.

### 4.3. Ovulation Time Has a Stronger Impact on the Overall Vitamin Stores Than Long-Term Feeding of Broodfish Feed in Sea Water

Manipulating the fish to ovulate earlier or later than normal (natural) came along with either a shorter or longer time to deposit nutrients in the eggs during gonad growth and maturation (vitellogenesis). Fjelldal et al. [4] reported larger eggs with higher dry matter from late ovulating females, which suggests an enhanced maternal investment and nutrient deposition with longer starvation time as indicated by emaciation and low viscera and hepatosomatic indexes in female broodfish. However, the manipulation of ovulation time also reduced egg quality [4].

This study found increased vitamin E deposition in eggs with time of ovulation, that is, higher levels from late compared to early or normal ovulating females. In contrast, the vitamin C content in the eggs from the late ovulation group was lowest compared to the other ovulation groups suggesting a protective role of vitamin C for vitamin E as observed for the broodfish tissues. Overall, the few significant differences in vitamin C and E concentrations between different feeding regimes may be due to the high dietary levels and the limited capacity to absorb excess of these vitamins.

The deposition of vitamin K1 in offspring followed the general nutrient deposition profile with the highest K1 stores in unfertilized eggs and fry from late ovulating females. The total MK4 stores, but not MK4 concentrations, were affected by ovulation timing. Interestingly, total MK4 stores increased over time in parallel with offspring development, while K1 stores declined—a trend observed in the data but not supported by statistical testing. This pattern may suggest a gradual conversion of K1 to MK4 during development [39]. A similar trend was observed in total whole body levels and gonadal concentrations, where MK4 deposition over time appeared more pronounced than that of K1, despite higher levels of K1 in the feed. While this pattern aligns with findings in mammals as described by Okano et al. [39], the specific metabolic pathways responsible for such conversion in fish remain poorly understood. To date, direct evidence for enzymatic conversion of K1 to MK4 in fish is lacking, which highlights the need for targeted studies on vitamin K metabolism in teleost species. Overall, few effects on K1 and MK4 levels in broodfish tissues by feeding regimes were observed.

The vitamin D3 status in broodfish was unaffected by 9 or 17 months of feeding the specialized broodfish feed in this study, likely due to similar D3 levels in both broodfish and grow-out feeds. However, females stored most vitamin D3 in their gonads when ovulating late after abstaining from feed for the longest time, while concentrations in the muscle from the same group diminished.

Overall, few effects of different feeding regimes (9 vs. 17 months) on vitamin deposition were observed, whereas ovulation manipulation, particularly late ovulation, had a more pronounced impact. That aligns with the conclusions of Fjelldal et al. [4] on the biological results, that feeding Atlantic salmon the broodfish feed for 9 months was sufficient, as extending the feeding period for 17 months offered few additional advantages. The observed changes in egg nutrient content and the effects of ovulation time manipulation on egg quality addressed by Fjelldal et al. call for re-evaluation of the procedures for Atlantic salmon broodstock husbandry.

Maternal deposition of vitamins into gonads and eggs is influenced by physiological and dietary factors [40, 41], and their persistence in early-stages depends on yolk utilization patterns and oxidative demands [42]. Yolk absorption rates and the timing of antioxidant depletion, particularly of vitamins E and C, can affect offspring skeletal development, immune competence, and oxidative stress resilience, highlighting an important avenue for future studies to optimize broodstock nutrition and offspring quality.

### 4.4. Are Vitamin Stores in the Gonads From Early and Late Ovulating Females Sufficient to Supply the Offspring Until First-Feeding?

For vitamin C and E, levels were very high in the feed, broodfish tissues and the offspring. Earlier requirement studies with first-feeding fry of Atlantic salmon reported that levels below 10 mg/kg for vitamin C, and below 15 mg/kg for vitamin E were associated with deficiency [36, 37]. The minimum requirements for vitamin C and E in salmon first-feeding fry has been set to 30 and 60 mg/kg dry feed, respectively [36, 37], as the requirement for vitamin E also greatly depends on other nutrients such as vitamin C, selenium, and polyunsaturated fatty acids [43]. Oxidation of the feed also increases the vitamin E requirement [28]. In the present study, the concentrations of both vitamins in first-feeding fry were above 40 mg/kg across all ovulation groups, which is well above the reported levels associated with deficiency and are, therefore, presumed to be sufficient to cover the needs at this stage. However, Hamre et al. [13] emphasized that excessive vitamin E intake may exert pro-oxidative effects, potentially causing oxidative stress rather than providing antioxidant benefits, particularly when unbalanced by other antioxidants, such as vitamin C, however, our study did not evaluate oxidative stress markers or antioxidant enzyme activity. Importantly, the normal ovulation group, which contained the same vitamin E concentrations as the early and late groups, did not exhibit increased deformity rates or reduced survival [4]. This suggests that vitamin E levels around 40 mg/kg in the fry were unlikely to have caused harmful effects at this developmental stage. Still, the threshold at which vitamin E shifts from antioxidant to pro-oxidant activity remains unclear.

Fewer requirement studies have been conducted on vitamin K in Atlantic salmon. One requirement study used increasing levels of the vitamin K derivative menadione nicotinamide bisulfite (MNB, vitamin K3) in the feed to first-feeding fry [29]. The authors did not observe any negative effects on blood coagulation, vertebra, and growth with approximately 4 µg/kg MK4 analyzed in fry at the zero dietary K3 inclusion. Here, salmon fry ready to first-feed were left with MK4 stores above 20 µg/kg and K1 stores above 4 µg/kg, which suggests that dietary vitamin K provided in all feeds was sufficient.

The vitamin D3 levels of the broodfish and grow-out feed batches (0.09–0.13 mg/kg) were above the estimated minimum requirements of 0.06 mg/kg for Atlantic salmon [38]. Two studies using 0.04 and 0.2 mg/kg vitamin D3 in the diet to salmon fry did not report any deficiency [44, 45]. In the present study, muscle and liver concentrations were relatively constant during feeding and starvation of broodfish, suggesting sufficient access to D3 from the feed. At the end, first-feeding fry holds on average 0.03 mg/kg of vitamin D3. However, it is difficult to conclude on the vitamin D3 status in the offspring as requirement studies on salmon first-feeding fry are lacking.

The four vitamins monitored in this study are unquestionably important for reproduction and larval development. The requirements for vitamin C and E, and most likely also vitamin K and D, were met for both broodfish and their offspring. A previous assessment of malformations in first-feeding fry showed no differences between the feeding and ovulation regimes [4]. However, late ovulating females produced larger, heavier eggs with higher dry matter content, but lower fertilization, hatching and survival rates compared to normal and early ovulation groups [4]. Reduced water content, higher concentrations of nutrients and other molecules, or additional physiological changes associated with the larger eggs may have contributed to these differences. The slightly lowered success of early ovulating females could be caused by the limited time for development and nutrient deposition prior to ovulation. It would be valuable to conduct follow-up studies on how manipulation of ovulation time and vitamin supplementation can affect different aspects of offspring quality, such as growth, robustness to handling, and disease resistance.

## 5. Conclusions

Muscle was the largest deposition tissue for the vitamins C, E, D, and K during the sea water growth period prior to gonadal maturation. Our findings show that ovulation timing had a greater impact on vitamin deposition in broodfish and transfer to eggs and fry than feeding duration. Specifically, delayed ovulation was associated with higher concentrations of the fat-soluble vitamins D3, E, and K1 in eggs and juveniles, potentially enhancing offspring nutrient density, while vitamin C levels were significantly lower in late ovulated eggs. Vitamin C and E levels, and most likely K and D in both broodfish and fry remained above known deficiency thresholds across all feeding and ovulation groups, supporting the adequacy of the feeding protocols used. However, late ovulation corresponded with reduced fertilization, hatching, and survival rates despite the production of larger and more nutrient-rich eggs. This indicates that factors beyond the content of the studied vitamins may influence reproductive success, highlighting the need for further research to optimize ovulation strategies that balance nutrient transfer and reproductive success in broodstock management.

## Figures and Tables

**Table 1:** Analyzed proximate composition and vitamin C, D, E, and K levels in grow-out and broodfish feed batches.

Nutrient		Grow-out feed	Grow-out feed	Broodfish feed	Broodfish feed	Broodfish feed
	Feed batch	G1	G2	B1	B2	B3
	Feeding period	Jan.20–May.20	May.20–Sep.20	Jan.20–May.20	May.20–Sep.20	Sep.20–Jun.21
Proximate composition						
Crude protein	g/100 g ww	39	38	39	39	38
Fat	g/100 g ww	35	33	32	32	33
Carbohydrates^a^	g/100 g ww	15.8	16.8	16.6	15.1	14.8
Dry matter	g/100 g ww	94	92	93	92	91
Ash	g/100 g ww	4.2	4.2	5.4	5.9	5.2
Vitamins						
Vitamin C (AAE)	mg/kg ww	760	180	610	580	550
Vitamin D3	mg/kg ww	0.12	0.13	0.13	0.12	0.09
Vitamin E (alpha-tocopherol)	mg/kg ww	340	250	580	450	430
Vitamin E (beta-tocopherol)	mg/kg ww	2.4	2	2.7	1.9	2.1
Vitamin E (gamma-tocopherol)	mg/kg ww	127	105	115	89	65
Vitamin E (delta-tocopherol)	mg/kg ww	15	11.7	24	15.3	14.4
Vitamin E (alpha-tocotrienol)	mg/kg ww	1.7	1.5	1.5	1.6	0.45
Vitamin E (beta-tocotrienol)	mg/kg ww	26	17.7	18.6	12.6	10.4
Vitamin E (gamma-tocotrienol)	mg/kg ww	3.7	3	<0.08	3.2	<0.08
Vitamin E (delta-tocotrienol)	mg/kg ww	<0.04	0.24	<0.04	0.22	<0.04
Vitamin K3 (menadione)	mg/kg ww	1.58	0.832	6.42	1.84	3.4
Vitamin K1	µg/kg ww	480	364	320	259	181
ß,γ-Dihydro vitamin K1	µg/kg ww	<1	<3.6	<1	<3.6	<2
Vitamin K2 (MK4)	µg/kg ww	6.3	6.6	11.2	15.1	18.5
Vitamin K2 (MK5)	µg/kg ww	<1	<7	<1	<9	<2
Vitamin K2 (MK6)	µg/kg ww	7.7	<6	10	<6	7.3
Vitamin K2 (MK7)	µg/kg ww	24.3	38.5	40.7	38.5	31.8
Vitamin K2 (MK8)	µg/kg ww	19.5	54.4	22.1	60.9	20
Vitamin K2 (MK9)	µg/kg ww	7	<4.8	10.7	<4.8	8.3
Vitamin K2 (MK10)	µg/kg ww	<1	<8.4	<1	<8.4	<4
Vitamin K (sum)^b^	µg/kg ww	544.8	463.5	414.7	373.5	266.9

*Note:* This table is modified from Fjelldal et al. [4] and a fully analyzed nutrient composition is provided in the supporting data in Supporting Information 1: Table S3.

Abbreviations: AAE, ascorbic acid equivalent; MK, menaquinone.

^a^Levels represent the subtracted values of protein, fat, and ash from dry matter content.

^b^Sum of vitamin K1 and K2 forms.

## Data Availability

The data that support the findings of this study are available in the Supporting Information.

## Supporting Information

Supporting Information 1 Table S1. Metadata on ovulating females and offspring. Table S2. Diet formulations. Table S3. Analyzed diet compositions. Figure S1. Vitamin C and E in broodfish. Figure S2. Vitamin K1 and K2 in broodfish. Figure S3. Vitamin D3 in broodfish. Figure S4. Vitamin C, E, K1, K2, and D3 in offspring stages.

Supporting Information 2 Table S4. Raw vitamin data.

Supporting Information 3 Table S5. Descriptive vitamin data and statistics.
