# Supplementary material for: Manipulated Spawning Along With an Extension of the Atlantic Salmon Broodfish Feeding Period Affect the Vitamin C, E, D, and K Status of Broodfish, Eggs, and First-Feeding Fry
Source: Aquac Nutr. 2025 Nov 11;2025:8874795. doi: 10.1155/anu/8874795 (PMC12626704; doi:10.1155/anu/8874795)
Supplement: Supporting Information 1 — Table S1. Metadata on ovulating females and offspring. Table S2. Diet formulations. Table S3. Analyzed diet compositions. Figure S1. Vitamin C and E in broodfish. Figure S2. Vitamin K1 and K2 in broodfish. Figure S3. Vitamin D3 in broodfish. Figure S4. Vitamin C, E, K1, K2, and D3 in offspring stages. [file 8874795.f1.pdf]

Supplementary Material 1

Manipulated spawning along with an extension of the Atlantic salmon broodfish feeding period affect the vitamin C, E, D and K status of broodfish, eggs and first-feeding fry

*Anne-Catrin Adam [*1], Per Gunnar Fjelldal [2], Tom Hansen [2], Ernst Morten Hevrøy [3] and Kristin Hamre [1]*

[*] Corresponding author: anne-catrin.adam@hi.no

[1] Feed and Nutrition, The Institute of Marine Research, Bergen, Norway

[2] Reproduction and Developmental Biology, The Institute of Marine Research, Matredal, Norway

[3] Mowi Feed, Mowi ASA, Bergen, Norway

Table S1. Metadata on ovulating females, eyed eggs and first-feeding juveniles.
DD, degree-days. First published in Fjelldal et al. (2024).

					Matre research station, IMR, Norway					
					Starvation time from on-land transfer until stripping	Stripping	Eyed eggs transport		First-feeding fry	
Ovulating female ID	Ovulation group	On-land tank ID	Feeding regime	Sea cage ID	Sum days	Date	Date	DD	Date	DD
**T1**	early	1	9 mths	12	153	01.11.2021	14.12.2021	374	16.02.2022	850
**T2**	early	3	9 mths	12	153	01.11.2021	14.12.2021	374	16.02.2022	850
**T3**	early	1	17 mths	11	153	01.11.2021	14.12.2021	374	16.02.2022	850
**T4**	early	3	17 mths	9	160	08.11.2021	21.12.2021	370	21.02.2022	828
**T5**	early	3	9 mths	12	160	08.11.2021	21.12.2021	370	21.02.2022	828
**T6**	early	1	9 mths	12	160	08.11.2021	21.12.2021	370	21.02.2022	828
**T7**	early	1	17 mths	9	160	08.11.2021	21.12.2021	370	21.02.2022	828
**T8**	early	1	17 mths	11	160	08.11.2021	21.12.2021	370	21.02.2022	828
**T9**	early	2	9 mths	10	160	08.11.2021	21.12.2021	370	21.02.2022	828
**T10**	early	3	9 mths	10	167	15.11.2021	28.12.2021	361	04.03.2022	852
**T11**	early	1	9 mths	10	167	15.11.2021	28.12.2021	361	04.03.2022	852
**T12**	early	3	17 mths	9	167	15.11.2021	28.12.2021	361	04.03.2022	852
**T13**	early	2	9 mths	10	167	15.11.2021	28.12.2021	361	04.03.2022	852
**T14**	early	2	9 mths	12	167	15.11.2021	28.12.2021	361	04.03.2022	852
**T15**	early	2	17 mths	9	167	15.11.2021	28.12.2021	361	04.03.2022	852
**T16**	early	2	9 mths	12	167	15.11.2021	28.12.2021	361	04.03.2022	852
**T17**	early	1	17 mths	9	167	15.11.2021	28.12.2021	361	04.03.2022	852
**T18**	early	(3)1	17 mths	11	181	29.11.2021	11.01.2022	334	21.03.2022	865
**T19**	early	1	9 mths	(10)12	181	29.11.2021	11.01.2022	334	21.03.2022	865
**T20**	early	2	17 mths	11	188	06.12.2021	18.01.2022	328	25.03.2022	838
**T21**	early	(3)2	9 mths	10	188	06.12.2021	18.01.2022	328	25.03.2022	838
**N1**	normal	5	9 mths	12	181	29.11.2021	11.01.2022	334	21.03.2022	865
**N2**	normal	4	17 mths	11	181	29.11.2021	11.01.2022	334	21.03.2022	865
**N3**	normal	5	17 mths	11	181	29.11.2021	11.01.2022	334	21.03.2022	865
**N4**	normal	6	17 mths	11	181	29.11.2021	11.01.2022	334	21.03.2022	865
**N5**	normal	6	17 mths	9	181	29.11.2021	11.01.2022	334	21.03.2022	865
**N6**	normal	5	17 mths	11	188	06.12.2021	18.01.2022	328	25.03.2022	838
**N7**	normal	6	17 mths	9	188	06.12.2021	18.01.2022	328	25.03.2022	838
**N8**	normal	4	17 mths	9	188	06.12.2021	18.01.2022	328	25.03.2022	838
**N9**	normal	6	9 mths	10	188	06.12.2021	18.01.2022	328	25.03.2022	838
**N10**	normal	6	9 mths	12	188	06.12.2021	18.01.2022	328	25.03.2022	838
**N11**	normal	6	9 mths	10	188	06.12.2021	18.01.2022	328	25.03.2022	838
**N12**	normal	4	9 mths	10	188	06.12.2021	18.01.2022	328	25.03.2022	838
**N13**	normal	6	17 mths	11	188	06.12.2021	18.01.2022	328	25.03.2022	838
**N14**	normal	4	9 mths	10	188	06.12.2021	18.01.2022	328	25.03.2022	838
**N15**	normal	5	9 mths	10	188	06.12.2021	18.01.2022	328	25.03.2022	838
**N16**	normal	5	17 mths	9	188	06.12.2021	18.01.2022	328	25.03.2022	838
**N17**	normal	5	9 mths	10	195	13.12.2021	31.01.2022	373	04.04.2022	859
**N18**	normal	4	9 mths	12	195	13.12.2021	31.01.2022	373	04.04.2022	859
**N19**	normal	4	17 mths	11	195	13.12.2021	31.01.2022	373	04.04.2022	859
**N20**	normal	4	17 mths	9	195	13.12.2021	31.01.2022	373	04.04.2022	859
**N21**	normal	5	9 mths	12	204	22.12.2021	07.02.2022	350	11.04.2022	838
**N22**	normal	6	9 mths	12	204	22.12.2021	07.02.2022	350	11.04.2022	838
**N23**	normal	4	9 mths	12	204	22.12.2021	07.02.2022	350	11.04.2022	838
**N24**	normal	5	17 mths	9	204	22.12.2021	07.02.2022	350	11.04.2022	838
**S1**	late	7	9 mths	12	244	31.01.2022	21.03.2022	384	20.05.2022	840

**S2**	late	9	17 mths	11	244	31.01.2022	21.03.2022	384	20.05.2022	840
**S3**	late	9	17 mths	9	244	31.01.2022	21.03.2022	384	20.05.2022	840
**S4**	late	7	17 mths	9	244	31.01.2022	21.03.2022	384	20.05.2022	840
**S5**	late	8	17 mths	11	244	31.01.2022	21.03.2022	384	20.05.2022	840
**S6**	late	9	9 mths	12	251	07.02.2022	24.03.2022	356	25.05.2022	834
**S7**	late	8	9 mths	12	251	07.02.2022	24.03.2022	356	25.05.2022	834
**S8**	late	9	17 mths	9	251	07.02.2022	24.03.2022	356	25.05.2022	834
**S9**	late	8	17 mths	9	251	07.02.2022	24.03.2022	356	25.05.2022	834
**S10**	late	8	17 mths	9	251	07.02.2022	24.03.2022	356	25.05.2022	834
**S11**	late	7	9 mths	10	251	07.02.2022	24.03.2022	356	25.05.2022	834
**S12**	late	8	9 mths	12	251	07.02.2022	24.03.2022	356	25.05.2022	834
**S13**	late	7	9 mths	12	251	07.02.2022	24.03.2022	356	25.05.2022	834
**S14**	late	9	17 mths	11	251	07.02.2022	24.03.2022	356	25.05.2022	834
**S15**	late	8	9 mths	10	251	07.02.2022	24.03.2022	356	25.05.2022	834
**S16**	late	7	17 mths	9	251	07.02.2022	24.03.2022	356	25.05.2022	834
**S17**	late	(8)7	9 mths	10	258	14.02.2022	04.04.2022	388	02.06.2022	847
**S18**	late	7	17 mths	11	258	14.02.2022	04.04.2022	388	02.06.2022	847
**S19**	late	7	17 mths	9	258	14.02.2022	04.04.2022	388	02.06.2022	847
**S20**	late	9	9 mths	10	258	14.02.2022	04.04.2022	388	02.06.2022	847
**S21**	late	9	9 mths	10	258	14.02.2022	04.04.2022	388	02.06.2022	847
**S22**	late	7	9 mths	10	258	14.02.2022	04.04.2022	388	02.06.2022	847
**S23**	late	8	17 mths	11	258	14.02.2022	04.04.2022	388	02.06.2022	847
**S24**	late	(9)8	9 mths	12	258	14.02.2022	04.04.2022	388	02.06.2022	847

Table S2. Diet formulation of grow-out diets and brood fish diets used during the experiment (all values are g/kg). Republished and modified table from Fjelldal et al. (2024).

	**G1**	**G2**	**B1**	**B2**	**B3**
Diet type	Grow-out diet	Grow-out diet	Brood fish diet	Brood fish diet	Brood fish diet
Feeding period	01/2020-5/2020	05/2020-9/2020	01/2020-5/2020	05/2020-9/2020	9/2020-6/2021
**Formulations**					
Fishmeal (Scandinavia)	98,9	78,2	252,7	250,9	271,2
Fishoil (Anchovetas)	25,2	18,8	-	-	8,0
Fishoil (Menhaden)	41,5	-	73,9	148,1	214,9
Fishoil (Scandinavia)	33,5	99,6	71,4	-	-
Rapeseed oil	217,2	220,1	145,3	148,1	74,3
Linseed oil	16,7	-	-	-	-
Soy protein concentrate	106,3	168,1	201,1	144,2	50,0
Corn gluten meal	52,0	51,5	-	50,5	-
Wheat gluten meal	149,9	160,1	34,7	72,3	160,0
Pea protein concentrate	38,4	-	46,6	-	-
Guar protein	52,0	25,8	-	-	-
Wheat whole grain	36,4	25,8	145,0	75,1	133,6
De-hulled horse beans	80,5	98,4	-	49,8	25,0
Amino acids, vitamins and minerals[1]	23,8	24,0	14,8	17,3	20,5
Pigment (10 % astaxanthin)	0,5	0,5	0,5	0,6	0,6
Feed phosphrous (MAP)	16,0	17,8	9,3	10,0	10,4
Water balance	9,6	10,3	4,2	32,9	31,5

Table S3. Analysed nutrient composition of five batches of grow-out and broodfish diet given to Atlantic salmon during the seawater period. Republished and modified table from Fjelldal et al. (2024).

		G1	G2	B1	B2	B3
	Feed type	Grow-out feed	Grow-out feed	Brood fish feed	Brood fish feed	Brood fish feed
	Feeding period	1/2020-5/2020	5/2020-9/2020	1/2020-5/2020	5/2020-9/2020	9/2020-6/2021
**Proximate composition**						
Crude protein	g/100g ww	39	38	39	39	38
Fat	g/100g ww	35	33	32	32	33
Carbohydrates [1]	g/100g ww	15,8	16,8	16,6	15,1	14,8
Dry matter	g/100g ww	94	92	93	92	91
Ash	g/100g ww	4,2	4,2	5,4	5,9	5,2
**Lipids**						
Cholesterol	mg/kg ww	716	1225	852	1862	1572
**Amino acids**						
Hydroxy-Proline	mg/g ww	<0.6	<0.6	1,7	1,5	1,5
Histidine	mg/g ww	8,3	7,5	8,5	7,6	7,7
Taurine	mg/g ww	0,75	0,6	1,52	1,25	1,93
Serine	mg/g ww	17,8	17,1	17,6	17,8	16,8
Arginine	mg/g ww	21,7	18,5	22,6	20,2	18,2
Glycine	mg/g ww	15,4	13,8	18,8	17,7	17,3
Aspartic acid	mg/g ww	29,1	27,8	36	33	28,1
Glutamic acid	mg/g ww	83	81	67	72	86
Threonine	mg/g ww	15,2	14,4	17	15,6	16,5
Alanine	mg/g ww	15,9	15,1	18,3	19,3	16,5
Proline	mg/g ww	26,7	26,2	21	22,4	26,4
Lysine	mg/g ww	22,6	22,2	25,3	26,3	25,3
Tyrosine	mg/g ww	13,3	11,4	12,2	12	11,7
Methionine	mg/g ww	8,8	7,8	8,9	8,6	8,9
Valine	mg/g ww	15,8	15,1	17,5	16,4	16,5
Isoleucine	mg/g ww	14,5	13,9	15,7	14,9	15,5
Leucine	mg/g ww	29,3	27,9	28,3	29,9	26,7
Phenylalanine	mg/g ww	18,7	16,7	17,6	16,8	18,7
**Fatty acids (mg/g ww)**						
06:0	mg/g ww	<0.01	<0.01	<0.01	<0.01	<0.01
08:0	mg/g ww	<0.01	<0.01	<0.01	<0.01	<0.01
10:0	mg/g ww	<0.01	<0.01	<0.01	<0.01	<0.01
12:0	mg/g ww	<0.01	<0.01	<0.01	<0.01	0,31
14:0	mg/g ww	7,75	6,33	12,04	12,91	21
14:1n-9	mg/g ww	<0.01	<0.01	0,28	<0.01	<0.01
15:0	mg/g ww	0,5	0,65	0,74	1,08	1,26
16:0	mg/g ww	24,99	27,59	28,3	34,48	43,24
16:1n-9	mg/g ww	<0.01	0,57	0,41	0,52	0,62
16:1n-7	mg/g ww	7,15	6,99	10,54	15,65	24,91
17:0	mg/g ww	0,53	0,91	0,84	1,45	1,44
16:2n-4	mg/g ww	0,32	1,31	1,12	2,5	4,01
18:0	mg/g ww	8,95	9,84	7,19	8	9,21
16:3n-3	mg/g ww	<0.01	<0.01	<0.01	<0.01	<0.01
18:1n-11	mg/g ww	0,49	0,67	0,72	0,53	0,31
18:1n-9	mg/g ww	108,03	133,76	80,36	85,47	49,06
18:1n-7	mg/g ww	12,28	8,55	9,11	8,39	8,21
16:4n-3	mg/g ww	<0.01	<0.01	<0.01	<0.01	<0.01
18:2n-6	mg/g ww	44,8	44,52	31,1	31,83	19,32
18:3n-6	mg/g ww	<0.01	<0.01	<0.01	0,38	0,61
20:0	mg/g ww	1,4	1,96	1,38	1,29	1,06
18:3n-3	mg/g ww	26,45	21,99	14,83	16,09	8,36
20:1n-11	mg/g ww	<0.01	0,55	0,68	<0.01	0,32
20:1n-9	mg/g ww	5,54	7,92	10,69	3,98	3,38
20:1n-7	mg/g ww	0,34	<0.01	<0.01	<0.01	0,54
18:4n-3	mg/g ww	2,4	3,04	4,31	3,22	5,3
20:2n-6	mg/g ww	<0.01	0,53	<0.01	0,36	0,35
20:3n-9	mg/g ww	<0.01	<0.01	<0.01	<0.01	<0.01
20:3n-6	mg/g ww	<0.01	<0.01	<0.01	0,32	0,47
22:0	mg/g ww	3,83	4,19	2,56	2,73	2,6
20:3n-3	mg/g ww	<0.01	<0.01	<0.01	<0.01	<0.01
20:4n-6 (ARA)	mg/g ww	0,62	1,1	0,99	1,96	2,41
22:1n-11	mg/g ww	4,67	7,56	14,57	1,73	2,39
22:1n-9	mg/g ww	1,32	1,63	1,71	1,1	0,65
20:4n-3	mg/g ww	0,99	0,67	1,47	1,68	2,56
20:5n-3 (EPA)	mg/g ww	10,83	10,07	14,23	16,49	31,35
24:0	mg/g ww	<0.01	0,37	<0.01	<0.01	<0.01
22:4n-6	mg/g ww	<0.01	<0.01	<0.01	<0.01	0,33
21:5n-3	mg/g ww	0,43	0,44	0,64	0,88	1,57
24:1n-9	mg/g ww	0,51	1,29	1,03	0,68	0,56
22:5n-6	mg/g ww	<0.01	<0.01	<0.01	0,64	0,68
22:5n-3 (DPA)	mg/g ww	1,34	0,96	1,9	3,32	5,23
22:6n-3 (DHA)	mg/g ww	7,41	12,47	12,15	13,57	19,66
24:5n-3	mg/g ww	<0.01	<0.01	<0.01	<0.01	<0.01
24:6n-3	mg/g ww	<0.01	<0.01	<0.01	<0.01	<0.01
Sum unidentified	mg/g ww	2,86	2,49	3,2	5,96	8,01

Sum identified	mg/g ww	285	320	268	275	274
Sum fatty acids	mg/g ww	288	322	271	281	282
Sum saturated	mg/g ww	47,9	51,8	53	62,1	80,1
Sum 16:1	mg/g ww	7,15	7,57	11	16,2	25,5
Sum 18:1	mg/g ww	121	143	90,2	94,4	57,6
Sum 20:1	mg/g ww	5,87	8,47	11,4	3,98	4,24
Sum 22:1	mg/g ww	5,99	9,19	16,3	2,83	3,04
Sum monounsaturated	mg/g ww	140	169	130	118	91,1
Sum EPA + DHA	mg/g ww	18,2	22,5	26,4	30,1	51
Sum n-3	mg/g ww	49,8	49,7	49,5	55,3	74
Sum n-6	mg/g ww	45,6	46,1	32,4	35,5	24,2
Sum polyunsaturated	mg/g ww	95,8	97,1	83	93,2	102
n-6/n-3	-	0,9	0,9	0,7	0,6	0,3
**Fatty acids (%)**						
06:0	%	<0.1	<0.1	<0.1	<0.1	<0.1
08:0	%	<0.1	<0.1	<0.1	<0.1	<0.1
10:0	%	<0.1	<0.1	<0.1	<0.1	<0.1
12:0	%	<0.1	<0.1	<0.1	<0.1	0,1
14:0	%	4,4	2,7	4,6	2	7,4
14:1n-9	%	0,1	<0.1	<0.1	<0.1	<0.1
15:0	%	0,3	0,2	0,4	0,2	0,4
16:0	%	10,4	8,7	12,3	8,6	15,3
16:1n-9	%	0,2	<0.1	0,2	0,2	0,2
16:1n-7	%	3,9	2,5	5,6	2,2	8,8
17:0	%	0,3	0,2	0,5	0,3	0,5
16:2n-4	%	0,4	0,1	0,9	0,4	1,4
18:0	%	2,7	3,1	2,9	3,1	3,3
16:3n-3	%	<0.1	<0.1	<0.1	<0.1	<0.1
18:1n-11	%	0,3	0,2	0,2	0,2	0,1
18:1n-9	%	29,7	37,5	30,4	41,5	17,4
18:1n-7	%	3,4	4,3	3	2,7	2,9
16:4n-3	%	<0.1	<0.1	<0.1	<0.1	<0.1
18:2n-6	%	11,5	15,6	11,3	13,8	6,8
18:3n-6	%	<0.1	<0.1	0,1	<0.1	0,2
20:0	%	0,5	0,5	0,5	0,6	0,4
18:3n-3	%	5,5	9,2	5,7	6,8	3
20:1n-11	%	0,3	<0.1	<0.1	0,2	0,1
20:1n-9	%	3,9	1,9	1,4	2,5	1,2
20:1n-7	%	<0.1	0,1	<0.1	<0.1	0,2
18:4n-3	%	1,6	0,8	1,1	0,9	1,9
20:2n-6	%	<0.1	<0.1	0,1	0,2	0,1
20:3n-9	%	<0.1	<0.1	<0.1	<0.1	<0.1
20:3n-6	%	<0.1	<0.1	0,1	<0.1	0,2
22:0	%	0,9	1,3	1	1,3	0,9
20:3n-3	%	<0.1	<0.1	<0.1	<0.1	<0.1
20:4n-6 (ARA)	%	0,4	0,2	0,7	0,3	0,9
22:1n-11	%	5,4	1,6	0,6	2,3	0,8
22:1n-9	%	0,6	0,5	0,4	0,5	0,2
20:4n-3	%	0,5	0,3	0,6	0,2	0,9
20:5n-3 (EPA)	%	5,2	3,8	5,9	3,1	11,1
24:0	%	<0.1	<0.1	<0.1	0,1	<0.1
22:4n-6	%	<0.1	<0.1	<0.1	<0.1	0,1
21:5n-3	%	0,2	0,2	0,3	0,1	0,6
24:1n-9	%	0,4	0,2	0,2	0,4	0,2
22:5n-6	%	<0.1	<0.1	0,2	<0.1	0,2
22:5n-3 (DPA)	%	0,7	0,5	1,2	0,3	1,9
22:6n-3 (DHA)	%	4,5	2,6	4,8	3,9	7
24:5n-3	%	<0.1	<0.1	<0.1	<0.1	<0.1
24:6n-3	%	<0.1	<0.1	<0.1	<0.1	<0.1
Sum unidentified	%	1,2	1	2,1	0,8	2,8
Sum identified	%	98,8	99	97,9	99,2	97,2
Sum fatty acids	%	100	100	100	100	100
Sum saturated	%	19,6	16,6	22,1	16,1	28,4
Sum 16:1	%	4	2,5	5,8	2,3	9
Sum 18:1	%	33,3	42	33,6	44,3	20,4
Sum 20:1	%	4,2	2	1,4	2,6	1,5
Sum 22:1	%	6	2,1	1	2,8	1,1
Sum monounsaturated	%	48	48,8	42	52,6	32,3
Sum EPA + DHA	%	9,7	6,3	10,7	7	18,1
Sum n-3	%	18,3	17,3	19,7	15,4	26,2
Sum n-6	%	12	15,8	12,6	14,3	8,6
Sum polyunsaturated	%	30,6	33,3	33,2	30,1	36,2
n-6/n-3	-	0,7	0,9	0,6	0,9	0,3
**Vitamins**						
Biotin (B7)	mg/kg ww	0,78	0,76	1	1	1,3
Cobalamin (B12)	mg/kg ww	0,3	0,24	0,25	0,41	0,34
Folate (B9)	mg/kg ww	6	9,1	7,4	15	16
Niacin (B3)	mg/kg ww	140	130	190	190	160
Pantothenic acid (B5)	mg/kg ww	54	58	85	89	89
Pyridoxine (B6)	mg/kg ww	15	14	23	22	25
Riboflavin (B2)	mg/kg ww	17	20	25	30	25
Thiamine -HCl (B1) [2]	mg/kg ww	25	24	37	43	31
Vitamin C equivalent (AAE)	mg/kg ww	760	180	610	580	550
Vitamin D3	mg/kg ww	0,12	0,13	0,13	0,12	0,09
Vitamin E (alpha-tocopherol)	mg/kg ww	340	250	580	450	430
Vitamin E (beta- tocopherol)	mg/kg ww	2,4	2	2,7	1,9	2,1

Vitamin E (gamma- tocopherol)	mg/kg ww	127	105	115	89	65
Vitamin E (delta- tocopherol)	mg/kg ww	15	11,7	24	15,3	14,4
Vitamin E (alpha- tocotrienol)	mg/kg ww	1,7	1,5	1,5	1,6	0,45
Vitamin E (beta- tocotrienol)	mg/kg ww	26	17,7	18,6	12,6	10,4
Vitamin E (gamma- tocotrienol)	mg/kg ww	3,7	3	< 0.08	3,2	< 0.08
Vitamin E (delta- tocotrienol)	mg/kg ww	< 0.04	0,24	< 0.04	0,22	< 0.04
Vitamin K3 (menadione)	mg/kg ww	1,58	0,832	6,42	1,84	3,4
Vitamin K (sum) [3]	µg/kg ww	544,8	463,5	414,7	373,5	266,9
Vitamin K1	µg/kg ww	480	364	320	259	181
ß,Y-Dihydro vitamin K1	µg/kg ww	<1	<3.6	<1	<3.6	<2
Vitamin K2 (MK4)	µg/kg ww	6,3	6,6	11,2	15,1	18,5
Vitamin K2 (MK5)	µg/kg ww	<1	<7	<1	<9	<2
Vitamin K2 (MK6)	µg/kg ww	7,7	<6	10	<6	7,3
Vitamin K2 (MK7)	µg/kg ww	24,3	38,5	40,7	38,5	31,8
Vitamin K2 (MK8)	µg/kg ww	19,5	54,4	22,1	60,9	20
Vitamin K2 (MK9)	µg/kg ww	7	<4.8	10,7	<4.8	8,3
Vitamin K2 (MK10)	µg/kg ww	<1	<8.4	<1	<8.4	<4
**Minerals**						
Ca	mg/kg ww	3600	3800	7700	11000	9100
Na	mg/kg ww	1600	1400	4100	3800	4200
K	mg/kg ww	6000	7200	7500	8200	5400
Mg	mg/kg ww	1300	1500	1700	1700	1400
P	mg/kg ww	8400	9200	9300	11000	9100
**Trace elements**						
Iodine	mg/kg ww	2,4	2	1,3	2,9	3,3
V	mg/kg ww	0,38	0,29	0,29	0,45	0,34
Cr	mg/kg ww	0,37	0,2	0,35	0,22	0,24
Mn	mg/kg ww	54	39	39	37	34
Fe	mg/kg ww	230	200	180	190	140
Co	mg/kg ww	0,17	0,16	0,11	0,13	0,12
Ni	mg/kg ww	0,69	0,49	0,42	0,47	<0.3
Cu	mg/kg ww	9,8	7,7	6,8	7,1	6,2
Zn	mg/kg ww	190	130	150	140	130
As	mg/kg ww	1,7	1,8	2,2	4,1	4,9
Se	mg/kg ww	0,59	0,62	0,92	0,93	0,97
Mo	mg/kg ww	2	1,4	1,3	0,93	0,61
Ag	mg/kg ww	<0.009	<0.008	<0.008	<0.008	<0.01
Cd	mg/kg ww	0,06	0,05	0,12	0,074	0,061
Hg	mg/kg ww	0,009	0,009	0,042	0,048	0,027
Pb	mg/kg ww	0,034	0,024	0,023	0,024	0,025

[1] Levels represent the subtracted values of protein, fat and ash from dry matter content.

[2] Hydrochloride (HCl) makes up 10,7% in thiamine -HCl.

[3] Sum of all vitamin K1 and K2 forms.

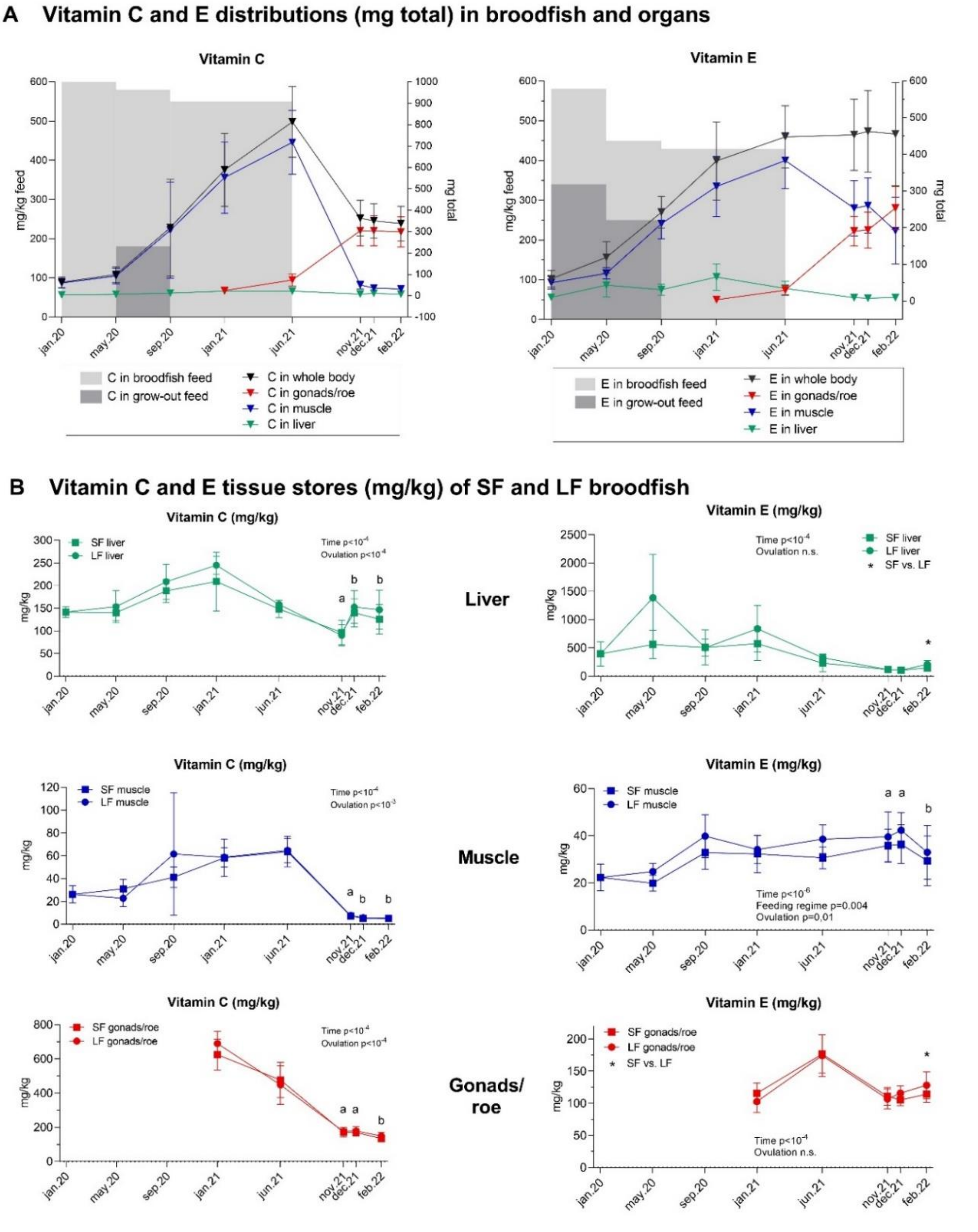

Figure S1. Vitamin C and E (α-tocopherol) levels in broodfish during different feeding regimes in the sea water period and during early, normal, and late ovulation. A: Dietary levels of each vitamin are illustrated in the background of each graph as grey shaded areas separating the levels for grow-out and broodfish feed (left y-axis: mg/kg feed), and the distribution of total vitamin amounts in liver, gonads, and muscle (right y-axis: mg total). The sum of each vitamin in the three organs gives an estimate of total vitamin in the whole body. Absent grey shaded areas in the background represent the transfer from sea-cages to on-land tanks and start of the starvation period (jun.21) in three different groups to ovulate either early, normal or late. Vitamin levels for each organ are presented as mean±SD of all groups and not separated by either feeding regimes or ovulation groups for clarity purposes. B: Figures illustrate vitamin C and E concentrations per organ (mg/kg) for both the SF (short-term feeding) and LF (long-term feeding) group following vitamin stores during the sea water and ovulation period. Vitamin levels are not separated by different curves after freshwater transfer (jun.21) to separate groups by ovulation time. An asterisk marks a significant difference between the feeding regimes at individual time points. Group means and statistics are provided in supplementary Table S5.

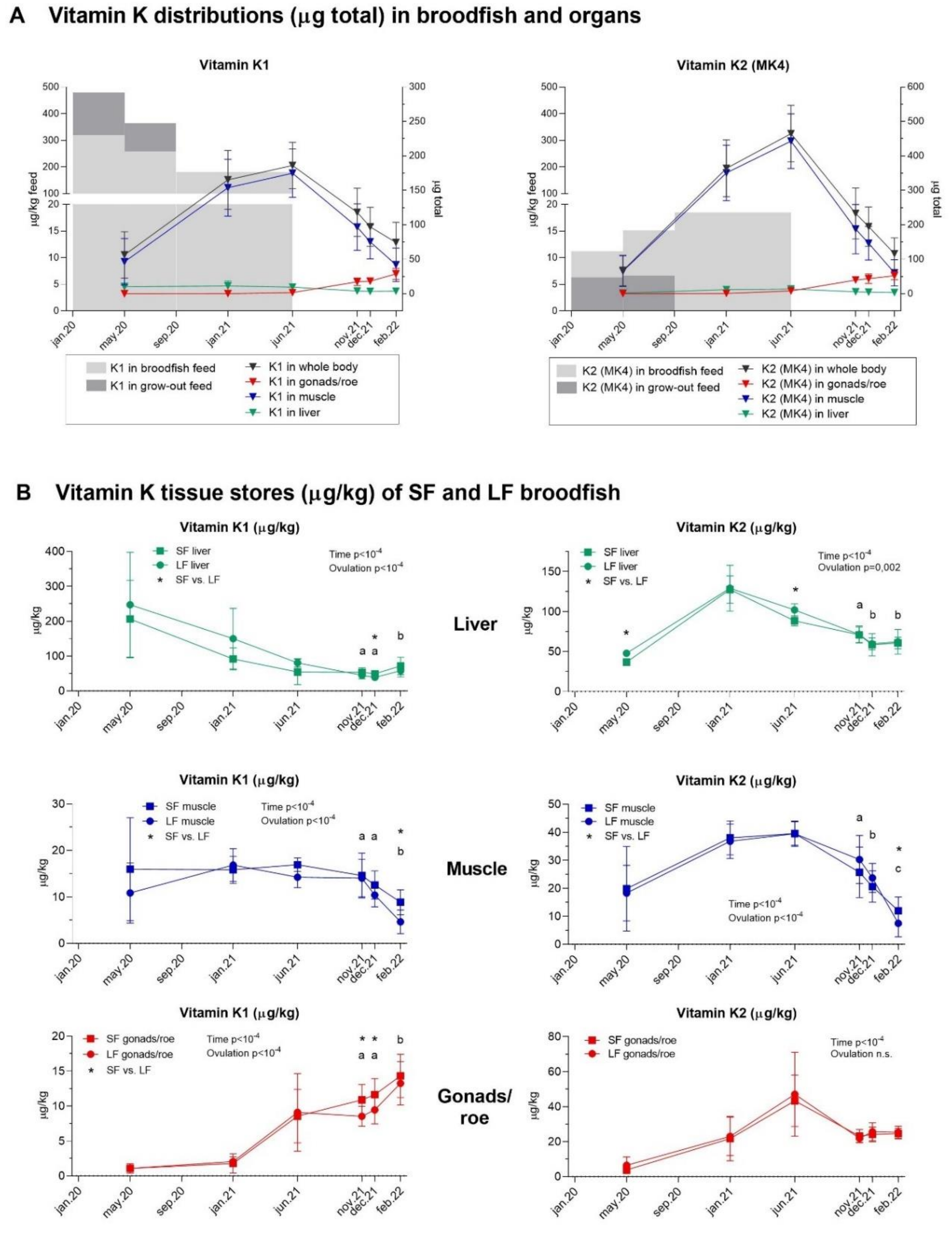

Figure S2. Vitamin K1 (phylloquinone) and K2 (menaquinone 4) levels in broodfish during the sea water period with different feeding regimes and during early, normal, and late ovulation. A: Dietary levels of both vitamin K forms are illustrated in the background of each graph as grey shaded areas separating the levels for grow-out and broodfish feed (left y-axis, µg/kg feed), and the distribution of total vitamin K in liver, gonads and muscle. The sum of vitamin K in the three organs gives an estimate of total vitamin K in the whole body (right y-axis, µg total). The sum of vitamin K in the three organs gives an estimate of total vitamin in the whole body. Absent grey shaded areas in the background represent the transfer from sea-cages to on-land tanks and start of the starvation period (jun.21) in three different groups to ovulate either early, normal or late. Vitamin K levels for each organ are presented as mean±SD of all groups and not separated by either feeding regimes or ovulation groups for clarity purposes. B: Figures illustrate vitamin K1 and K2 (MK4) concentrations per organ (µg/kg) for both the SF (short-term feeding) and LF (long-term feeding) groups following vitamin stores during the sea water and ovulation period. Vitamin levels are not separated by different curves after freshwater transfer (jun.21) to separate groups by ovulation time. An asterisk marks a significant difference between the feeding regimes at individual time points. Group means and statistics are provided in supplementary Table S5.

A Vitamin D3 distributions (mg total) in broodfish and organs

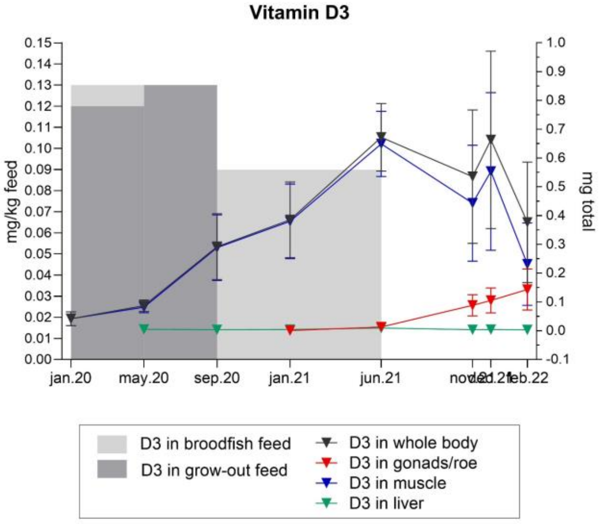

B Vitamin D3 tissue stores (mg/kg) of SF and LF broodfish

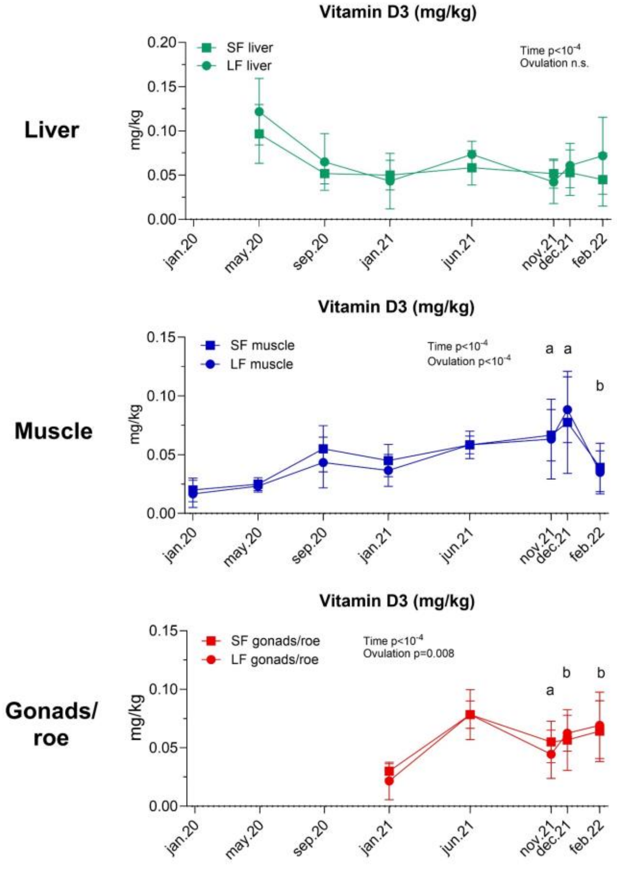

Figure S3. Vitamin D3 (cholecalciferol) levels in broodfish during the sea water period with different feeding regimes and during early, normal, and late ovulation. A: Dietary levels of vitamin D3 are illustrated in the background of each graph as grey shaded areas separating the levels for grow-out and broodfish feed (left y-axis, mg/kg feed), and the distribution of total vitamin amounts presented in liver, gonads, muscle and whole body (right y-axis, mg total). Total levels in broodfish represent the sum of liver, muscle, and gonad levels (mg total) at each timepoint. The sum of vitamin D3 in the three organs gives an estimate of total vitamin in the whole body. Absent grey shaded areas in the background represent the transfer from sea-cages to on-land tanks and start of the starvation period (jun.21) in three different groups to ovulate either early, normal or late. Vitamin D3 levels for each organ are presented as mean±SD of all groups and not separated by either feeding regimes or ovulation groups for clarity purposes. **B:** Figures illustrate vitamin D3 concentrations per organ (mg/kg) for both the SF (short-term feeding) and LF (long-term feeding) group following vitamin stores during the sea water and ovulation period. Vitamin levels are not separated by different curves after freshwater transfer (jun.21) to separate groups by ovulation time. An asterisk marks a significant difference between the feeding regimes at individual time points. Group means and statistics are provided in supplementary Table S5.

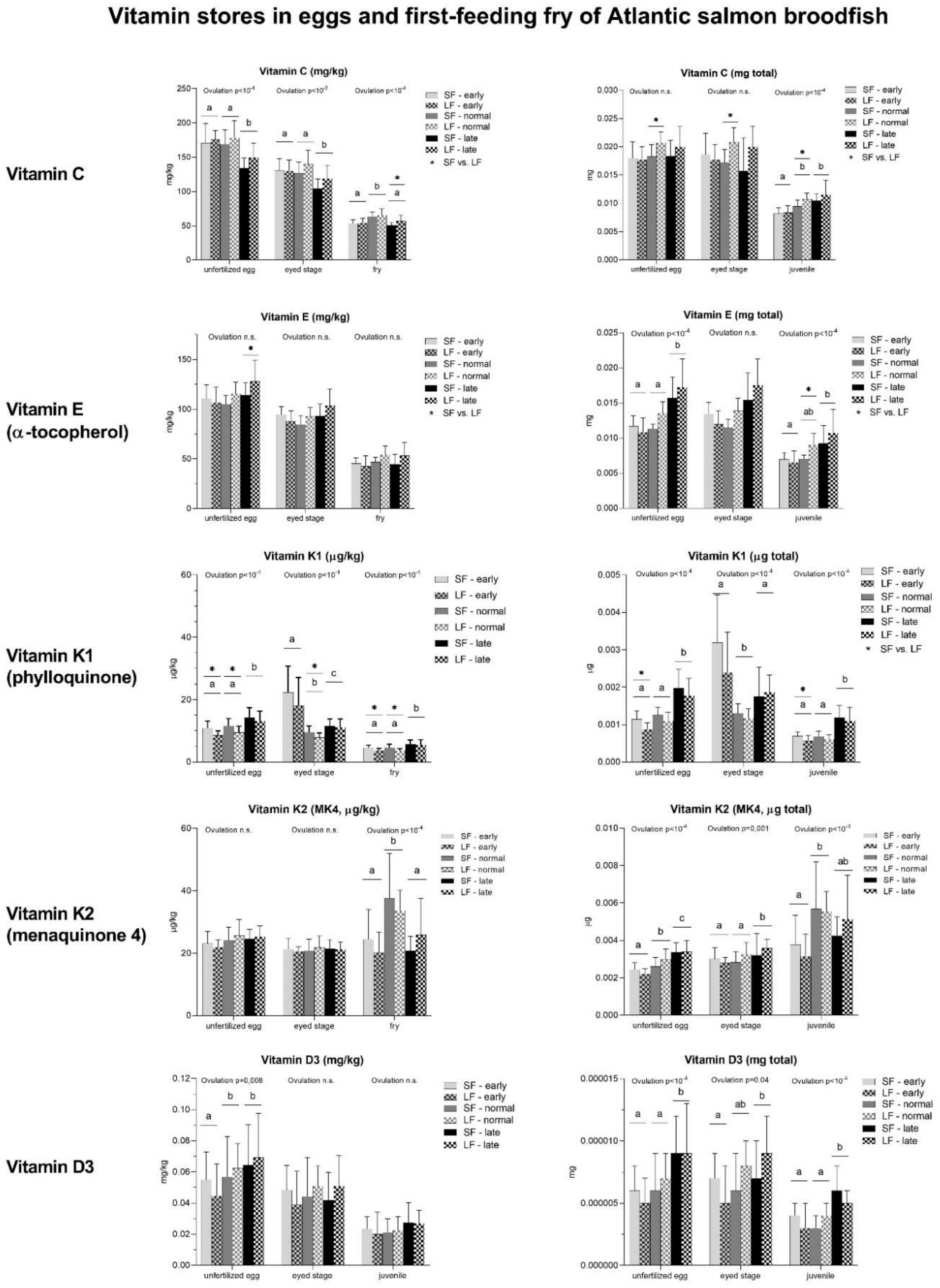

Figure S4. Vitamin C, E, K and D concentrations and total content analyzed in unfertilized eggs, eyed eggs, and first-feeding fry depending on the broodfish feeding regime and early, normal, or late ovulation. Vitamin levels given as mean±SD at each stage from early, normal, and late ovulating broodfish that have been fed a specialized broodfish feed for 17 (LF) or 9 months (SF). Concentrations (mg/kg or µg/kg) presented for unfertilized eggs correspond to the reported gonad concentrations at time of ovulation. Significant ovulation group differences are indicated by lower case letters and an asterisk marks significant feeding regime differences at individual time points. Group means and statistics are provided in supplementary Table S5.
